# A near-infrared Sn-Pb perovskite imager with monolithic integration

**DOI:** 10.1038/s41377-025-02127-y

**Published:** 2026-01-20

**Authors:** Ciyu Ge, Chengjie Deng, Jiaxing Zhu, Yongcheng Zhu, Qi Xu, Borui Jiang, Long Chen, Yuxuan Liu, Boxiang Song, Ping Fu, Chao Chen, Liang Gao, Jiang Tang

**Affiliations:** 1 Wuhan National Laboratory for Optoelectronics (WNLO) and School of Optical and Electronic Information (SOEI), Huazhong University of Science and Technology, Wuhan, China; 2 State Key Laboratory of Pulsed Power Laser Technology, Hefei, China; 3 Optics Valley Laboratory, Wuhan, China; 4 Wenzhou Advanced Manufacturing Technology Research Institute of Huazhong University of Science and Technology, Wuhan, China; 5 JFS Laboratory, Wuhan, China; 6 Key Laboratory of Photoelectric Conversion and Utilization of Solar Energy, Dalian Institute of Chemical Physics, Chinese Academy of Sciences, Dalian, China; 7 Shenzhen Huazhong University of Science and Technology Research Institute, Wuhan, China

**Keywords:** Photonic devices, Imaging and sensing, Optoelectronic devices and components

## Abstract

Solution-processed Sn-Pb perovskites have emerged as promising candidates for near-infrared (NIR) photodetectors due to their low-cost, tunable bandgap and scalable fabrication. However, Sn^2+^ oxidation creates Sn vacancies and undesirable p-type doping, resulting in high dark current and limited detectivity, which hinder the practical deployment of Sn-Pb perovskite photodetectors. Herein, we propose a Sn(SCN)_2_ inorganic molecular surface passivation strategy to suppress Sn^2+^ oxidation, significantly reduce surface defect density and enhance the optoelectronic properties (a dark current density of 10 nA cm^−2^ at a bias of −0.1 V and a high specific detectivity of ~1.6 × 10^13^ Jones). Leveraging this approach, we report the monolithically integrated Sn-Pb perovskite NIR imager with a complementary metal-oxide-semiconductor readout circuit. The imager, featuring a 640 × 512 pixel array with a 15 μm pixel pitch, achieves an external quantum efficiency of 76% at 940 nm and a modulation transfer function of 206.5 LW/PH at 50%. Furthermore, the Sn-Pb perovskite imager demonstrates advanced material recognition capabilities, including liquid identification, underscoring its potential in chemical sensing, biomedical imaging and industrial inspection.

## Abstract (web summary)

Monolithic Sn-Pb perovskite NIR imager with Sn(SCN)_2_ passivation achieves low dark current, high detectivity and material recognition for advanced sensing and imaging.

## Graphical abstract

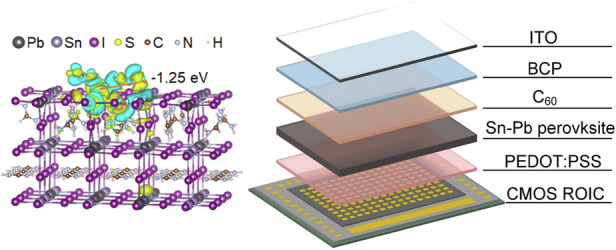

## Introduction

Photodetectors, which convert incident light signals into modulated electrical signals, are critical components in modern optoelectronics^1–5^. Particularly, near-infrared (NIR) photodetectors afford for widespread applications in security screening^6,7^, material identification^8^, machine vision^9,10^ and autonomous driving^11,12^. Recently, emerging photodetectors based on organic semiconductors and quantum dots have gained significant attention due to their tunable bandgaps and low-temperature solution-processability. However, the external quantum efficiency (EQE) in the NIR region remain significantly lower than in the visible spectrum (typically below 60%)^13–15^.

Halide perovskites, with high absorption coefficient, high carrier mobility, low production cost and compatibility with solution processing, have been widely explored for photovoltaics^16,17^, displays^18–24^ and photodetection^25–27^ applications. Replacing part of Pb in Pb-based perovskites with Sn has been adopted to extend the absorption cut-off edge to ~1000 nm, making Sn-Pb perovskites as promising candidates for NIR photodetectors^28,29^. However, the development of Sn-Pb perovskite-based photodetectors has been limited by the high dark current density and low specific detectivity (D*)^30–35^. Though the low-temperature solution processing enables monolithic integration with silicon-based readout integrated circuit (ROIC) to form NIR imager, there is still no publicly reported NIR imager based on Sn-Pb perovskite up to now. Indeed, the integration of perovskite with ROIC has been successfully applied to both visible and X-ray imagers^36–39^.

One of the main challenges is the delocalized 5s^2^ lone pair electrons of Sn^2+^ make it susceptible to be oxidized into Sn^4+^, leading to the detrimental Sn vacancies (V_Sn_) and undesirable p-type doping^40–42^. Many studies have introduced antioxidants or strong complexing agents (e.g., SnF_2_^43^, Sn powder^44^, tin acetate (Sn(Ac)_2_^45^)) into Sn-Pb perovskite precursors to mitigate bulk oxidation. Choy et al., introduced Sn(SCN)_2_ into the Sn-Pb perovskite precursor, utilizing the pseudo-halide SCN^−^ to regulate the crystallization processes and improve the performance of NIR photodetectors^46^. However, the surface of Sn-Pb perovskites remains prone to oxidation during subsequent device fabrication and operation. Consequently, the defect state density at the upper interface of Sn-Pb perovskites is three orders of magnitude higher than that in the bulk^47,48^. Although some studies have successfully suppressed the dark current density by optimizing the electron transport layer, this often comes at the cost of reduced responsivity and EQE, leading to a lower D^*^^49^. Suppressing surface oxidation and reducing interface defect density are key to simultaneously minimizing dark current density and enhancing the detectivity of Sn-Pb perovskite photodetectors and imagers.

In this work, we propose a surface passivation strategy using Sn(SCN)_2_ molecular crystals to address these challenges. Density functional theory (DFT) calculations reveal that the Sn^2+^ in Sn(SCN)_2_ preferentially interact with V_Sn_ on the perovskite surface and is adsorbed via coordination bonding, effectively passivating the surface defects. The resulting Sn-Pb perovskite photodetectors exhibit an ultralow dark current density (10 nA cm^−2^ at −0.1 V) and a high D^*^ (~1.6 × 10^13^ Jones). We firstly report a NIR imager that monolithically integrates Sn-Pb perovskite with a larger-scale ROIC made using complementary metal–oxide–semiconductor (CMOS) around the world. The imager are integrated with a CMOS ROIC of 640 × 512 pixel array (15 μm pixel pitch) shows an EQE of 76% at 940 nm and an MTF50 (modulation transfer function at 50% contrast) of 206.5 LW/PH. Our imager demonstrates the liquid identification capabilities, showing it can be used for matter identification.

## Results

### Interfacial passivation mechanisms on Sn-Pb perovskite film

**Fig. 1:**
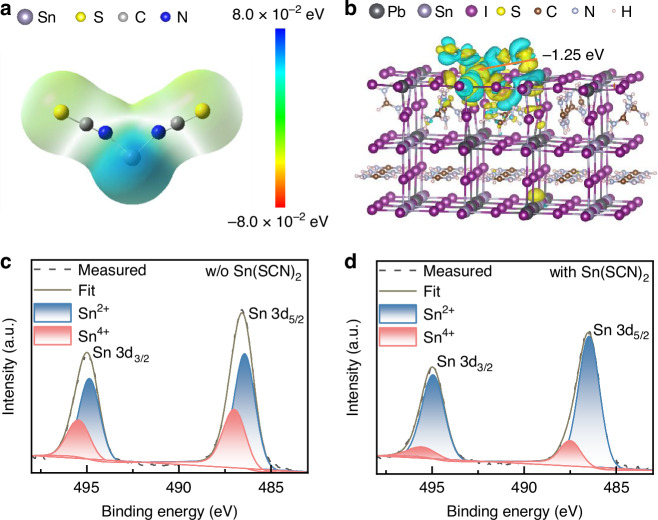
Material properties and interconnection mechanism between Sn(SCN)_2_**and perovskite**. **a** Structure and ESP of Sn(SCN)_2_. **b** Atomic structure and adsorption energy of Sn(SCN)_2_ absorbed on the perovskite surface. **c**, **d** Sn 3 d XPS spectra of Sn-Pb perovskite films

We designed and synthesized Sn(SCN)_2_ molecules^50^ (supplementary Note 1, Fig. S1, S2) to effectively passivate V_Sn_ in Sn-Pb perovskite interface. We analyzed the charge distribution of Sn(SCN)_2_ using molecular electrostatic potential (ESP) mapping (Fig. 1a), identifying the Sn^2+^ ions in Sn(SCN)_2_ as potential interaction sites with the surface of Sn-Pb perovskites. Furthermore, we calculated the adsorption energy of Sn(SCN)_2_ molecules on the Sn-Pb perovskite surface (Fig. 1b), obtaining a value of −1.25 eV. Fourier transform infrared (FTIR) spectra reveal C = N stretching peak shifts from 1712.30 cm^−1^ to 1711.67 cm^−1^ after Sn(SCN)_2_ passivation (Fig. S3), indicating an interaction between Sn(SCN)_2_ and the perovskite surface. These results demonstrate that Sn(SCN)_2_ can adsorb onto the Sn-Pb perovskite surface via coordination bonding, filling V_Sn_ and mitigating surface defects.

Additionally, Sn(SCN)_2_, as a molecular crystal, features significant steric hindrance between adjacent Sn^2+^ ions, making the formation of Sn(SCN)_4_ or other Sn^4+^-based oxidation products unlikely. This property is critical for suppressing the oxidation of Sn^2+^ on the surface of Sn-Pb perovskites. The Sn 3 d X-ray photoelectron spectroscopy (XPS) spectra of the Sn-Pb perovskite films (Fig. 1c, d) confirmed that Sn(SCN)_2_ effectively inhibits the oxidation of Sn^2+^. Notably, the Sn^4+^ content decreased significantly from 34.71% to 13.01%. Ultraviolet photoelectron spectroscopy (UPS) reveals that Sn(SCN)_2_ passivation raises the Fermi level of the perovskite film (Fig. S4a). Correspondingly, the near surface electron concentration increases from 1.1 × 10^10 ^cm^−3^ to 7.6 × 10^10 ^cm^−3^ (Fig. S4b), providing strong evidence for the effective reduction of surface V_Sn_. Therefore, the use of Sn(SCN)_2_ not only passivates V_Sn_ on the Sn-Pb perovskite surface but also suppresses the oxidation of surface Sn^2+^, ultimately reducing the surface defect density.

### Improved Sn-Pb perovskite film quality and underlying mechanism

**Fig. 2:**
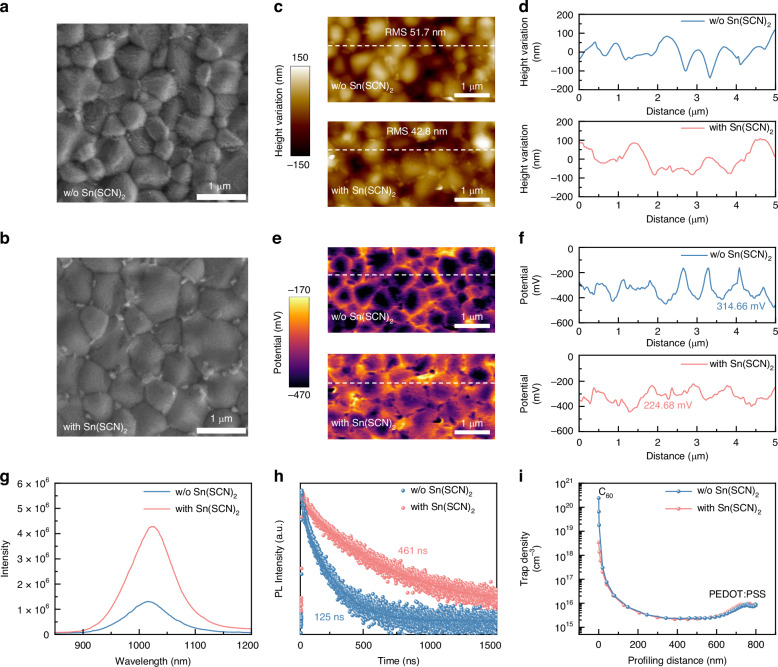
Passivation of Sn-Pb perovskite film surface with Sn(SCN)_**2**_. **a**, **b** Top-view SEM images of Sn-Pb perovskite films. **c** AFM images of Sn-Pb perovskite films. **d** Profiling surface roughness along the white dash line in (**c**). **e** KPFM images of Sn-Pb perovskite films. **f** Profiling surface potential along the white dash line in (**e**). **g** Steady-state PL spectra of Sn-Pb perovskite films deposited on bare glass. **h** TRPL spectra of Sn-Pb perovskite films deposited on bare glass. **i** DLCP measurements for Sn-Pb perovskite films

Furthermore, we characterized and analyzed Sn-Pb perovskite films before and after Sn(SCN)_2_ treatment. Scanning electron microscopy (SEM) images (Fig. 2a, b) revealed that Sn(SCN)_2_ predominantly accumulates at the grain boundaries of the perovskite film, likely due to its primary role on passivating grain boundary defects. This treatment effectively fills voids at the grain boundaries, reducing the surface roughness from 51.7 nm to 42.8 nm and resulting in a smoother and more uniform film morphology (Fig. 2c, d).

Kelvin probe force microscopy (KPFM) results further confirmed a reduction in the overall surface potential and a more uniform potential distribution (Fig. 2e). The dashed lines in Fig. 2e represent the horizontal surface potential distribution, further detailed in Fig. 2f. After Sn(SCN)_2_ treatment, the potential difference decreases significantly from 314.66 mV to 224.68 mV, reflecting improved uniformity of the surface potential. These results suggest that Sn(SCN)_2_ effectively passivates surface defects, indicating a lower work function and reduced p-type self-doping. These findings are consistent with the observed the decreased Sn^4+^ content as shown in Fig. 1d.

Figure 2g, h show the steady-state photoluminescence (PL) spectra and time-resolved photoluminescence (TRPL) decays of the Sn-Pb perovskite films, respectively. After Sn(SCN)_2_ treatment, the Sn-Pb perovskite film exhibits significantly enhanced PL intensity, and its carrier lifetime increases from 125 ns to 461 ns. Furthermore, we compared the defect distribution in perovskite films via drive-level capacitance profiling (DLCP, Fig. 2i). The results revealed that the defect density in the bulk and at the lower interface of the perovskite films remains nearly unchanged, but the defect density at the upper interface decreases by two orders of magnitude after Sn(SCN)_2_ treatment. Time-of-flight secondary ion mass spectrometry (ToF-SIMS) analysis provides additional evidence that Sn(SCN)_2_ mainly interacts with the surface of the Sn-Pb perovskite film (Fig. S5). The findings align well with the PL and TRPL observations, indicating that Sn(SCN)_2_ treatment effectively suppresses nonradiative recombination at the film surface and reduces surface defects.

### Performance of Sn-Pb perovskite photodetector

**Fig. 3:**
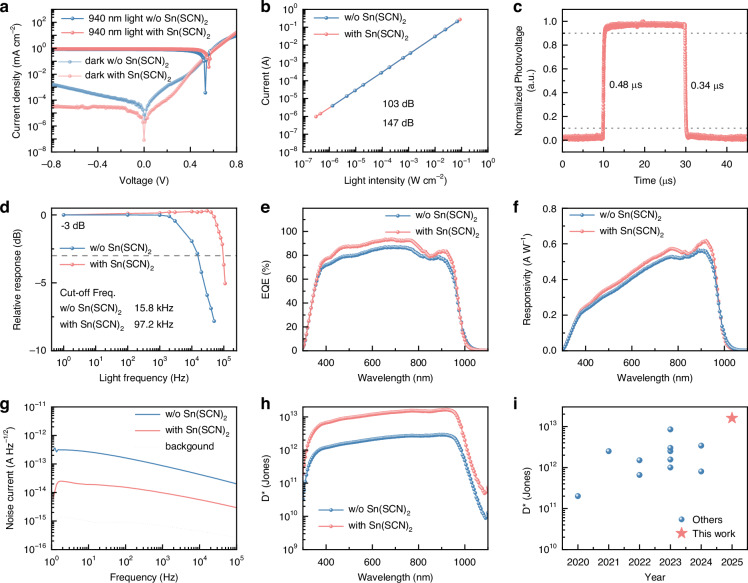
Performance of CMOS-compatible Sn-Pb perovskite photodetectors. **a** Current density versus voltage curves of Sn-Pb perovskite photodetectors under dark and 940 nm light with a power density of 500 µW/cm^2^. **b** LDR of the Sn-Pb perovskite photodetectors. **c** Transient response of Sn-Pb perovskite photodetectors with Sn(SCN)_2_ at zero bias. **d** Response bandwidth of Sn-Pb perovskite photodetectors at zero bias. **e** EQE of Sn-Pb perovskite photodetectors. **f** Responsivity spectra of Sn-Pb perovskite photodetectors. **g** Measured noise current of Sn-Pb perovskite photodetectors at zero bias. **h** Detectivity versus wavelength of Sn-Pb perovskite photodetectors. **i** Specific detectivity over 900 nm of Sn-Pb perovskite photodiodes reported in the literatures

We fabricated Sn-Pb perovskite photodetectors with the structure of ITO/PEDOT:PSS/Sn-Pb perovskite film/C_60_/BCP/Ag. After Sn(SCN)_2_ treatment on the upper interface of the Sn-Pb perovskite film, the dark current density of the photodetector significantly decreases with improved uniformity (Figs. 3a, S6). At a bias of −0.1 V, the dark current density reduces from 61 nA cm^−2^ to 10 nA cm^−2^, which can be attributed to lower defect state density at the film surface. We also measured the photocurrent density curve under 940 nm monochromatic light with a power density of 500 µW/cm^2^, and the detector with Sn(SCN)_2_ exhibited a higher photocurrent density. Under varying light intensities from ~0.3 μW to 90 mW, the photocurrent is linearly proportional to the light intensity. Notably, in the self-powered state (zero bias), the Sn(SCN)_2_-treated Sn-Pb perovskite photodetector achieves a linear dynamic range (LDR) of 147 dB, comparable to that of silicon photodiodes.

We tested the transient response of the photodetector after Sn(SCN)_2_ treatment. In the self-powered state, the rising and falling times of the device are 0.48 μs and 0.34 μs, respectively (Fig. 3c). Compared to the photodetector without Sn(SCN)_2_ treatment (Fig. S7), the device with Sn(SCN)_2_ exhibits a faster response speed and lower parasitic capacitance during charging and discharging, benefiting from effective passivation of interface defects. Additionally, the frequency response of the device demonstrates a − 3 dB bandwidth of up to 97.2 kHz (Fig. 3d), echoing the observed transient response. The responsivity slightly increases with the light modulation frequency, attributed to the discharge of junction capacitance. This phenomenon has also been observed in PbS quantum dots, Si/Ge and InGaAs photodiodes^51,52^. This rapid photoresponse is adequate to support typical imaging applications running at 30 frames per second^53^.

EQE spectra indicate that the Sn-Pb perovskite photodetector exhibits excellent photoresponse across a broad wavelength range of 300–1000 nm (Fig. 3e). After Sn(SCN)_2_ treatment, the photodetector achieves a higher EQE of 76% at 940 nm. This improvement is attributed to the reduced defect state density at the perovskite film surface. Additionally, the Sn(SCN)_2_-treated photodetector demonstrates a higher responsivity (R), achieving 0.58 A/W at 940 nm under a self-powered mode (Fig. 3f).

Figure 3g presents the frequency-dependent current noise spectra at zero bias, directly measured using a preamplifier and lock-in amplifier. Notably, the background noise limit is lower than that of the devices. The photodetector treated with Sn(SCN)_2_ exhibits a noise current (*i*_n_) an order of magnitude lower than that of the control device, measuring only ~9.76 fA/Hz^−1/2^ at 1 kHz. This substantial reduction in noise current suggests that our approach effectively suppresses 1/*f* noise at high frequency, making it particularly advantageous for imaging applications. We selected the measured noise current at 1 kHz to calculate D* to ensure consistency with the responsivity measurement conditions. The D^*^ can be calculated according to the equation,

1 $${D}^{* }=\frac{R\sqrt{\mathrm{A\varDelta }f}}{{i}_{n}}$$

where *A* is the device area (0.04 cm^2^), Δ*f* is the electrical bandwidth as 1 Hz here. The D* of Sn(SCN)_2_-treated Sn-Pb perovskite photodetector achieves ~1.6 × 10^13^ Jones, which is 13 times higher than the control photodetector (Fig. 3h) and comparable to a commercial InGaAs photodiode^54^ (around 4 × 10^12^ Jones). Among all the reported Sn-Pb perovskite photodiodes, our device simultaneously achieves the highest D* and the lowest dark current density (Fig. 3i). We also measured the EQE, noise current, and D* at −0.1 V bias (Fig. S8). The results indicate that the variations in these key metrics between 0 V and −0.1 V are minimal. This is because Sn-Pb perovskites exhibit a strong built-in electric field, ensuring that the carrier collection efficiency remains nearly unchanged within this bias voltage range. It is worth noting that the passivation behavior of Sn(SCN)_2_ differs from our previous report using Pb(SCN)_2_ additives in the precursor solution. In that case, Pb(SCN) _2_ could be homogeneously incorporated into the bulk without hindering carrier transport, thereby reducing bulk defect density. We note that incorporating Sn(SCN)_2_ into the perovskite precursor can also effectively suppress the dark current density (21 nA cm^−2^ at −0.1 V, Fig. S9a). However, due to the intrinsically poor conductivity of Sn(SCN)_2_, its incorporation into the bulk phase hindered carrier transport, leading to reduced EQE in the resulting Sn-Pb perovskite detector (Fig. S9b), which is detrimental for imaging applications. Therefore, in this work, we focused on employing Sn(SCN)_2_ as a surface passivation strategy, which simultaneously achieves dark current suppression and maintains high EQE.

Additionally, the device exhibited good operational stability. The dark current density of devices with Sn(SCN)_2_ exhibited negligible variation after 20 days of storage (Fig. S10a). In contrast, devices without Sn(SCN)_2_ displayed an increase of nearly two orders of magnitude at −0.1 V (Fig. S10b). For Sn(SCN)_2_-passivated devices, the dark current density rose to about twice its initial value after storage at 65 °C in nitrogen, in stark contrast to the ~15-fold increase observed in devices without passivation (Fig. S10c, d). Moreover, the devices with Sn(SCN)_2_ retained stable dark current density after 24 h continuous working a bias of −0.1 V, while the control devices exhibited a fivefold increase (Fig. S11). The unencapsulated device operated continuously in air for over 1300 seconds with almost no photocurrent degradation (Fig. S12). These advancements highlight that Sn(SCN)_2_ passivation effectively improves both storage and operation stability of unencapsulated devices and lay a solid foundation for the development of high-quality Sn-Pb perovskite imager integrated with CMOS ROIC.

### Performance of Sn-Pb perovskite imager

**Fig. 4:**
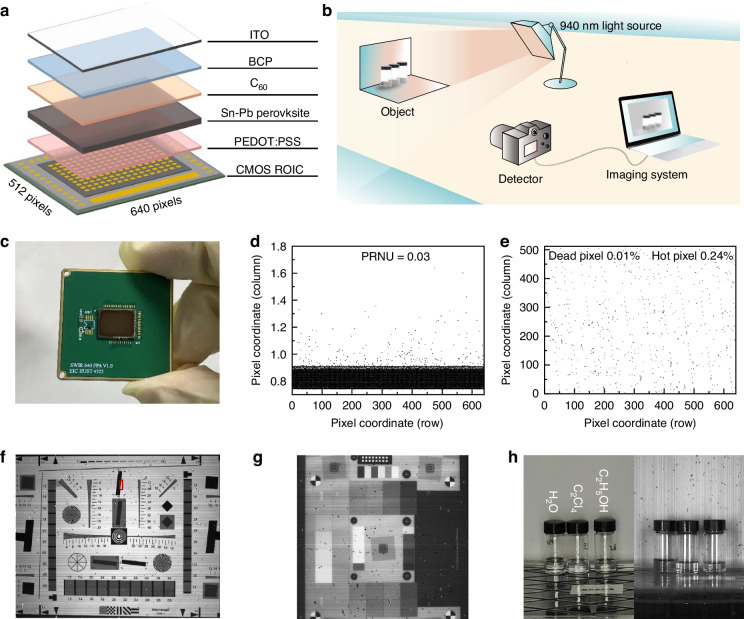
Performance of Sn-Pb perovskite imager. **a** Structure diagram of Sn-Pb perovskite imager. **b** Schematic diagram of the imaging processes. **c** Photograph of Sn-Pb perovskite imager. **d** Pixel grayscale distribution of Sn-Pb perovskite imager. **e** Dead pixels and hot pixels of the Sn-Pb perovskite imager. **f** ISO-12233 test chart imaged by the Sn-Pb perovskite imager with a 940 nm LED. **g** Dynamic range chart-36 imaged by the Sn-Pb perovskite imager with a 940 nm LED. **h** Photographs of H_2_O, C_2_Cl_4_ and C_2_H_5_OH captured by phone camera with a visible light (left) and Sn-Pb perovskite imager with a 940 nm LED (right)

Prior to testing the chip performance, we examined single-pixel devices with different electrode materials (Fig. S13). The measured dark current densities are nearly same, demonstrating that substituting ITO with Au on the PEDOT:PSS side has negligible impact on device performance. We fabricated Sn(SCN)_2_-treated Sn-Pb perovskite photodetectors layer by layer on a CMOS ROIC. The Sn-Pb perovskite imager with 640 × 512 pixels (15 μm pixel pitch) is schematically illustrated in Fig. 4a, with a photograph of the imager as shown in Fig. 4c. The microscopic image of Sn-Pb perovskite photodiodes covering on the CMOS ROIC chip is shown in Fig. S14a and the cross-section SEM image of the Sn-Pb perovskite imager is shown in Fig. S14b. The imaging system shown in Fig. 4b was built to capture NIR images using the Sn-Pb perovskite imager work at −0.1 V bias. Based on calculations in supplementary Note 2, the photoresponse non-uniformity (PRNU) of the Sn-Pb perovskite imager is 3% (Fig. 4d). To further evaluate the noise performance of the imager, we performed blackbody testing to obtain the statistical distribution of the noise voltage across the entire focal plane (Fig. S15). The results show that the noise follows an approximately Gaussian distribution, which ensures uniform pixel response and good imaging quality. Dead pixels are defined as those with signals below 50% of the average signal, while hot pixels are defined as those with effective noise voltages exceeding twice the mean noise voltage. Due to the superior detection performance of the Sn(SCN)_2_-treated Sn-Pb perovskite photodetectors, the perovskite imager exhibits an exceptionally low fraction of dead pixels (0.01%) and hot pixels (0.24%), demonstrating its excellent imaging quality (Fig. 4e).

ISO 12233 slanted-edge method was used to measure the spatial resolution. A standard test chart captured by the Sn-Pb perovskite imager under a 940 nm LED is shown in Figs. 4f, S16. The MTF was obtained by resolving the diagonal edges marked in the red rectangles. The MTF50 (50% contrast spatial frequency) value of the slant edge is 206.5 LW/PH (Fig. S17), demonstrating imaging performance comparable to that of the commercial InGaAs imager (Fig. S18). The LDR of the imager is 58.2 dB and the noise equivalent power is 16.4 ${\rm{fW}}/\sqrt{{Hz}}$ (supplementary Note 3). Due to its high LDR, the photograph of the Dynamic range chart-36 with 36 gray levels in Fig. 4g shows all the gray levels are clearly distinguished by the Sn-Pb perovskite imager.

We finally presented an application of this Sn-Pb perovskite imager. The left side of Fig. 4h shows images of three solvents (H_2_O, C_2_Cl_4_ and C_2_H_5_OH) captured under visible light, and the absorption spectra of the solvents are shown in Fig. S19a. All three appear as colorless and transparent liquids, making them difficult to distinguish under visible light. The right side of Fig. 4h presents NIR images of the same solvents captured by the Sn-Pb perovskite imager under 940 nm illumination. In these images, H_2_O appears darkest, C_2_H_5_OH lighter, and C_2_Cl_4_ the lightest, enabling clear differentiation between the three solvents (Fig. S19b–d). We included a video demonstration showcasing the imaging performance of our Sn-Pb perovskite NIR imager (Supplementary Video 2). The video clearly illustrates the absence of image lag, confirming that our reset mechanism effectively clears residual charges and ensures real-time imaging capability. We selected 15 frames in 7 s–7.5 s to identify the effect of residual shadow and found no obvious effect between images in each frame (Fig. S20). The imager without Sn(SCN)_2_ showed a significant decline in imaging quality after 7 days, and deteriorated severely after 14 days. In contrast, the imager with Sn(SCN)_2_ maintained good imaging performance even after 14 days, demonstrating its enhanced stability (Fig. S21). This imaging capability and material distinction highlight the broad application potential of the Sn-Pb perovskite imager in diverse scenarios.

## Discussion

We proposed an interface passivation strategy for Sn-Pb perovskite films by introducing Sn(SCN)_2_, achieving high D^*^ (~1.6 × 10^13^ Jones) and low current density (10 nA cm^−2^ at −0.1 V). Based on this, we developed the high-performance Sn-Pb perovskite imager with 640 × 512 pixels. We demonstrated the potential of these imagers for distinguishing H_2_O, C_2_Cl_4_ and C_2_H_5_OH, showcasing its versatility and application potential.

## Materials and methods

### Materials and solvents

The raw materials and solvents were not subjected to any purification. PbI_2_ (99.999%), SnI_2_ (99.999%), FAI ( > 99%) and MAI ( > 99%), were purchased from Advanced Election Technology Co., Ltd. Pb(SCN)_2_ (99.5%), CsI (99.999%), EDAI_2_ (99.5%), PEDOT:PSS and C_60_ were purchased from Xi’an Yuri Solar Co., Ltd. SnF_2_ (99%), Sn powder (99.999%), N,N-dimethylformamide (DMF, 99.8%, anhydrous), dimethyl sulfoxide (DMSO, 99.9%, anhydrous), chlorobenzene (CB, 99.9%, anhydrous) and isopropanol (IPA, 99.5%, anhydrous) were purchased from Sigma-Aldrich. Bathocuproine (BCP) was purchased from TCI. The glass ITO with a sheet resistance of 7 ~ 9 ohm/sq was purchased from Advanced Election Technology Co. Ltd.

### Precursor preparation

The Cs_0.1_FA_0.6_MA_0.3_Sn_0.5_Pb_0.5_I_3_ perovskite precursor with a concentration of 2.0 mol L^−1^ was prepared by mixing CsI (52.0 mg), FAI (206.3 mg), MAI (95.3 mg), SnI_2_ (372.6 mg), PbI_2_ (461.0 mg), SnF_2_ (15.7 mg) and Pb(SCN)_2_ (3 mg) in mixed solvents of 0.25 mL DMSO and 0.75 mL DMF. The precursor solution was filtered through a 0.22 μm PTFE filter before use.

### Theoretical calculation

All calculations in this study were performed with the Vienna ab initio Simulation Package (VASP)^55^ within the frame of density functional theory (DFT). The exchange-correlation interactions of electron were described via the generalized gradient approximation (GGA) with PBE functional^56^, and the projector augmented wave (PAW) method^57^ was used to describe the interactions of electron and ion. Additionally, the DFT-D3 method^58,59^ was used to account for the long-range van der Waals forces present within the system. The Monkhorst-Pack scheme^60^ was used for the integration in the irreducible Brillouin zone. The kinetic energy cut-off of 450 eV was chosen for the plane wave expansion. The lattice parameters and ionic position were fully relaxed, and the total energy was converged within 10^−5^ eV per formula unit. The final forces on all ions are less than 0.02/Å.

### Device fabrication

The Cs_0.1_FA_0.6_MA_0.3_Sn_0.5_Pb_0.5_I_3_ perovskite detectors have a device structure of ITO/PEDOT:PSS/perovskite/C_60_/BCP/Ag. The PEDOT:PSS was spin-coated on the ITO substrates at 2000 rpm for 30 s and annealed at 100 °C for 10 min in the air. After cooling, the Sn-Pb perovskite films were spin-coated onto the substrates at 4000 rpm for 8 s in a nitrogen glovebox. The wet Sn-Pb perovskite films were immediately transferred into a vacuum chamber (120 mL) for a pumping time of 20 s at a vacuum degree of ~1 Pa. Then, the Sn-Pb perovskite films were annealed under a light radiation annealing for 1 min as we previously reported^61^. After cooling, the Sn-Pb perovskite films were post-treated by spinning a solution of Sn(SCN)_2_ (0.5 mol mL^−1^) in a 1:1 IPA:CB solvent at 4000 rpm for 30 s and annealed at 100 °C for 5 min. Then, the substrates were transferred to the evaporative chamber inside the nitrogen glovebox. C_60_ (15 nm)/BCP (7 nm)/Ag (150 nm) were sequentially deposited on the top of the perovskite by thermal evaporation.

### Sn-Pb perovskite imager fabrication

The same Sn-Pb perovskite photodiodes were deposited directly on the pixel electrode array of the CMOS ROIC chip layer by layer. The hole transport layer, active layers and electron transport layers were prepared sequentially as described above, with the common pads covered by a tape. Then, a 200 nm ITO layer as a common top electrode was fabricated by magnetron sputtering in a DC mode at 100 W under an Ar atmosphere. Finally, the Sn-Pb perovskite imager was bonded to a metal casing in PGA format, providing a hermetic seal and electrical connection with the camera module.

### Device characterizations

The XPS spectra for perovskite films were conducted using the AXIS SUPRA+ instrument from Shimadzu-Kratos (Japan). The perovskite film morphology was imaged with a scanning electron microscope (SEM, FEI Navo NanoSEM450). The AFM and KPFM images were obtained in the ambient atmosphere using a Bruker Dimension Icon XR AFM. The steady-state PL was measured using a laser confocal Raman spectrometer (LabRAM HR800, Horiba JobinYvon) and the light was illuminated from both the front and back surface of the perovskite films (excited by 532 nm). The TRPL was measured using a spectrofluorometer (QuantaMaster 8000 series fluorometers, Horiba), and the samples were excited by a 532 nm pulsed laser. The DLCP test used the Agilent E4294A impedance analyzer. The scanning voltage offset range is −0.1 V to 0.8 V and the a.c. voltage frequency was set to 10 kHz. The current density-voltage (*J*-*V*) and current density-time (*J*-*t*) curves were tested by an Agilent B1500A semiconductor characterization system. The 940 nm light sources were Thorlabs monochromatic light LEDs modulated by a pulse function generator (Agilent 332100 A). The noise current spectra were collected on a dynamic signal analyzer (Agilent 35670 A). The signal was amplified using a low-noise current preamplifier (Stanford Research Systems, SR570). The noise spectra were measured from 100 Hz to 100 kHz using a semicon-ductor parameter analyzer (Platform Design AutomationFS380) with a noise module. EQE measurements were performed in ambient air using a QE system (EnliTech) with monochromatic light focused on a device pixel and a chopper frequency of 20 Hz. The stability test in air were conducted at a relative humidity of 40% ± 10% both 25 °C and 65 °C. Electrical aging is carried out by using the KEITHLEY 2450 source meter in a nitrogen atmosphere of 25 °C, and then using the Agilent B1500A to test and record the aging data. All the devices were evaluated in their unencapsulated state. The images of the gray-scale were illuminated in 800 K blackbody radiation to ensure sufficiently strong and uniform light. The camera framing and focal length setting of the lens were under ISO test standards.

## Supplementary information

Supplementary Information

the video of the Sn-Pb perovskite NIR imager

## Data Availability

The main data in this study are provided in the Supplementary Information. The data that support the findings of this study are available from the corresponding author upon reasonable request.
