# Supplementary material for: A near-infrared Sn-Pb perovskite imager with monolithic integration
Source: Light Sci Appl. 2026 Jan 20;15:73. doi: 10.1038/s41377-025-02127-y (PMC12815914; doi:10.1038/s41377-025-02127-y)
Supplement: Supplementary file 1 — Supplementary Information [file 41377_2025_2127_MOESM1_ESM.pdf]

A near-infrared Sn-Pb perovskite imager with monolithic integration

Ciyu Ge[1], Chengjie Deng[1,2], Jiaxing Zhu[1], Yongcheng Zhu[1], Qi Xu[1], Borui Jiang[1], Long Chen[1,3], Yuxuan Liu[4], Boxiang Song[1,3,5], Ping Fu[6], Chao Chen[1,3,*], Liang Gao[1,2,3,4,7*], Jiang Tang[1,3,*]

[1] Wuhan National Laboratory for Optoelectronics (WNLO) and School of Optical and Electronic Information (SOEI), Huazhong University of Science and Technology, Wuhan, 430074, China.

[2] State Key Laboratory of Pulsed Power Laser Technology, Hefei 230037, China.

[3] Optics Valley Laboratory, Wuhan, 430074, China.

[4] Wenzhou Advanced Manufacturing Technology Research Institute of Huazhong University of Science and Technology, 325006, China.

[5] JFS Laboratory, Wuhan, 430078, China.

[6] Key Laboratory of Photoelectric Conversion and Utilization of Solar Energy, Dalian Institute of Chemical Physics, Chinese Academy of Sciences, Dalian, 116023

[7] Shenzhen Huazhong University of Science and Technology Research Institute, 518057, China.

[*] Corresponding author. Email: cchen@hust.edu.cn, highlight@hust.edu.cn, jtang@mail.hust.edu.cn

Supplementary Information

Supplementary Note 1

To synthesize $Sn(SCN)_2$, 10 mmol of $SnSO_4$ is dissolved in 18 mL of H_2O and 1 mL of H_2SO_4. Separately, 24 mmol of NaSCN is dissolved in 2 mL of H_2O to prepare a solution with a concentration slightly exceeding 12 M. The NaSCN solution is then added dropwise to the $SnSO_4$ solution, and the mixture is stored in a refrigerator for 6 hours. The resulting needle $Sn(SCN)_2$ crystals are collected by filtration and thoroughly washed with deionized water (Fig. S1).

Supplementary Note 2

Photoresponse non-uniformity (PRNU), a critical parameter for evaluating image sensors, is calculated based on the spatial variation of pixel responses under uniform illumination. The typically equation given by

$$\mathrm{PRNU} = \frac{1}{I}\sqrt{\frac{1}{N}\sum_{0}^{N-1}(I_i - \bar{I})^2} \tag{S1}$$

where $\bar{I}$ is the average photocurrent of the pixel, I_i is the photocurrent of the pixel number of i, and N is the number of pixels.

Supplementary Note 3

We measured the average noise voltage (V_{noise}) of the imaging chip as 1.2 mV, and the conversion gain of the existing circuit (g) as 2.13 μV/e[1]. The average noise electron number (σ_{noise}) is as below

$$\sigma_{noise} = \frac{V_{noise}}{g} = \frac{1.2\ mV}{2.13\mu V/e^-} \approx 564\ e^- \qquad \text{(S2)}$$

The noise equivalent power (NEP) is as below[2]

$$\text{NEP} = \frac{\sigma_{noise} \times q}{Rt} \qquad \text{(S3)}$$

where R is the responsivity, t is the integration time. According to the NEP formula, when the incident light is 940 nm, the corresponding NEP of single pixel is 16.4 fW/$\sqrt{Hz}$.

The LDR of the imaging module in CMOS circuit can be calculated as below[3]

$$\text{LDR} = 20\log(\text{FWC}/\sigma_{noise}) \qquad \text{(S4)}$$

where FWC is the full well capacity of the circuit (here is 460 ke$^-$)[4]. The LDR of the imaging module in CMOS is 58.2 dB.

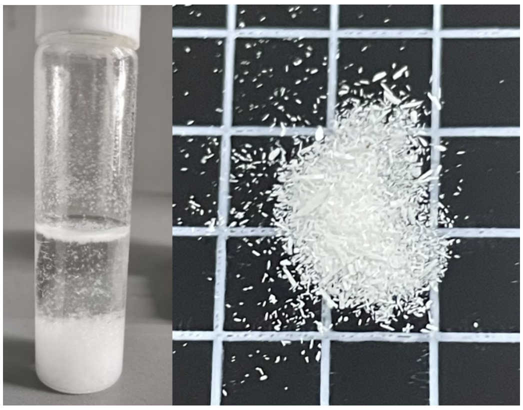

Fig. S1 | Photograph of synthesized $Sn(SCN)_2$ precipitate and powder.

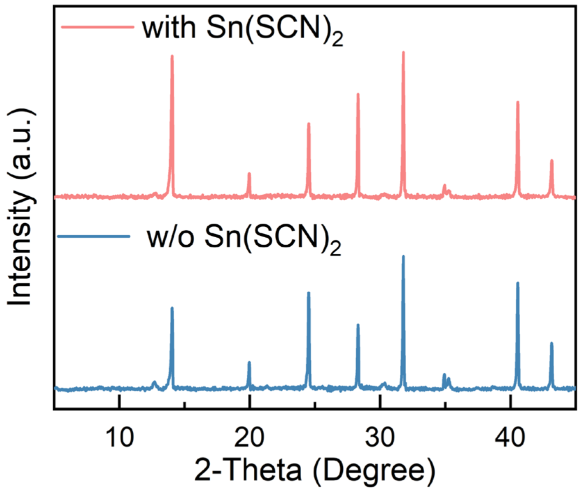

Fig. S2 | XRD patterns of Sn-Pb perovskite films with and without $Sn(SCN)_2$.

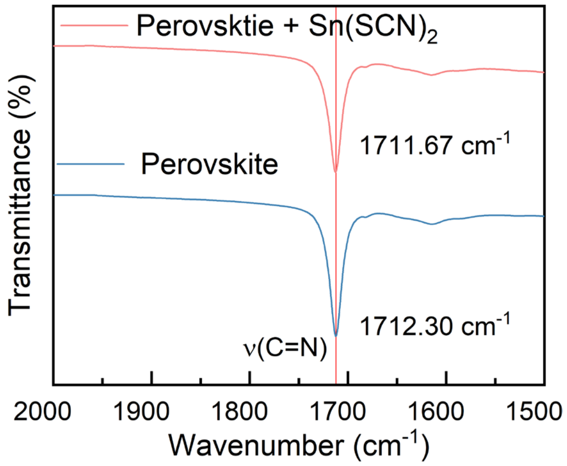

Fig. S3 | FTIR spectra of Sn-Pb perovskite film with and with $Sn(SCN)_2$ passivation.

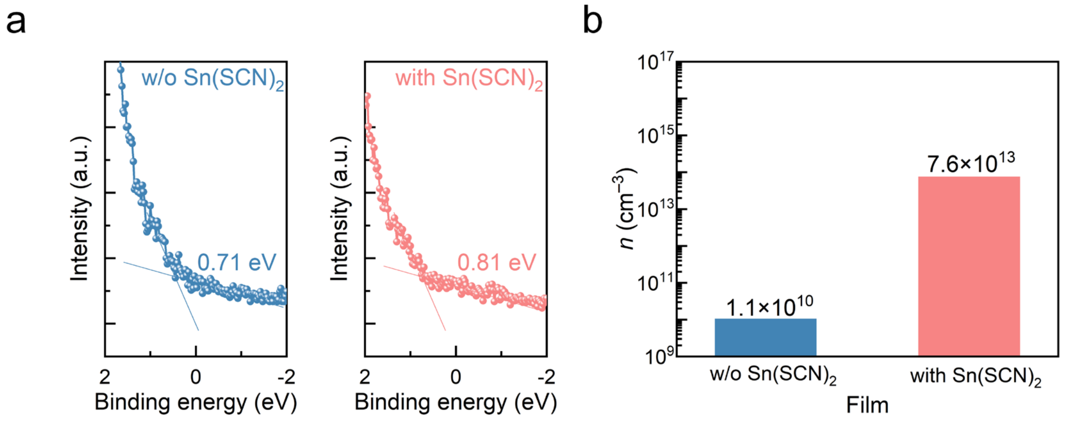

Fig. S4 | a UPS spectra of Sn-Pb perovskite films. **b** Electron density in the conduction band near the surface of the Sn-Pb perovskite films.

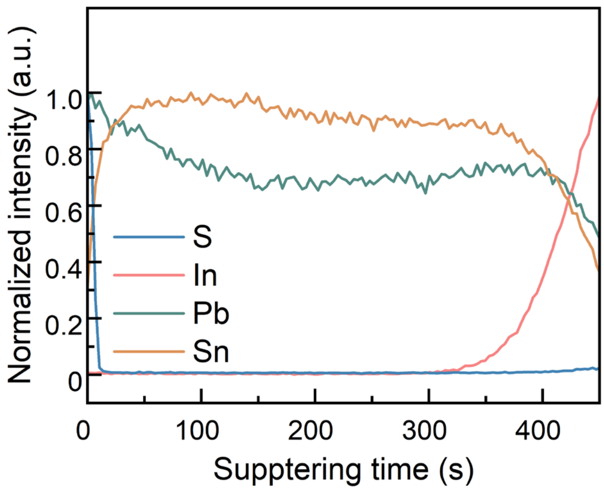

Fig. S5 | ToF-SIMS depth profiles showing elemental distribution of Sn-Pb perovskite films after $Sn(SCN)_2$ passivation.

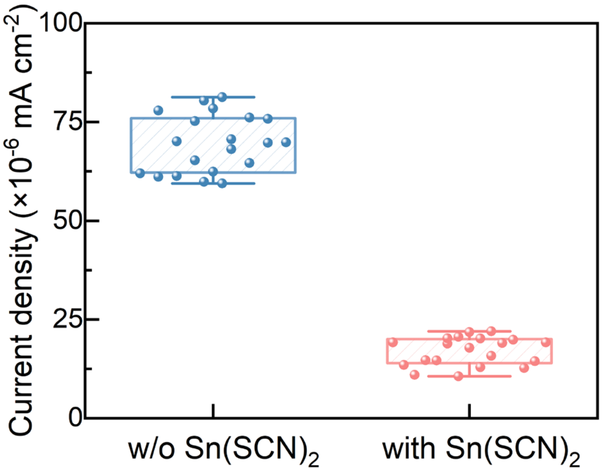

Fig. S6 | Statistical distribution of dark current density for twenty devices without $Sn(SCN)_2$ and twenty devices with $Sn(SCN)_2$ passivation.

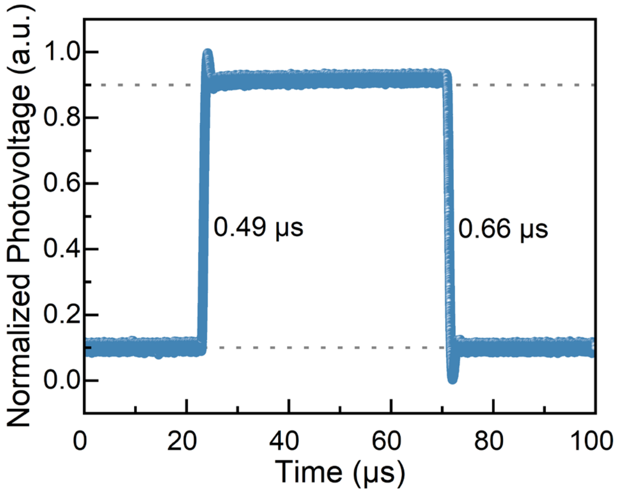

Fig. S7 | Transient response of Sn-Pb perovskite photodetectors without $Sn(SCN)_2$ at zero bias.

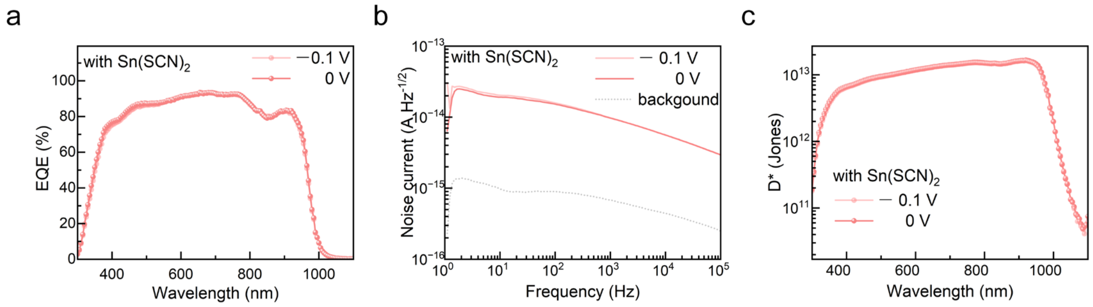

Fig. S8 | a EQE, **b** measured current noise and **c** detectivity versus wavelength of Sn-Pb perovskite photodetectors at different bias.

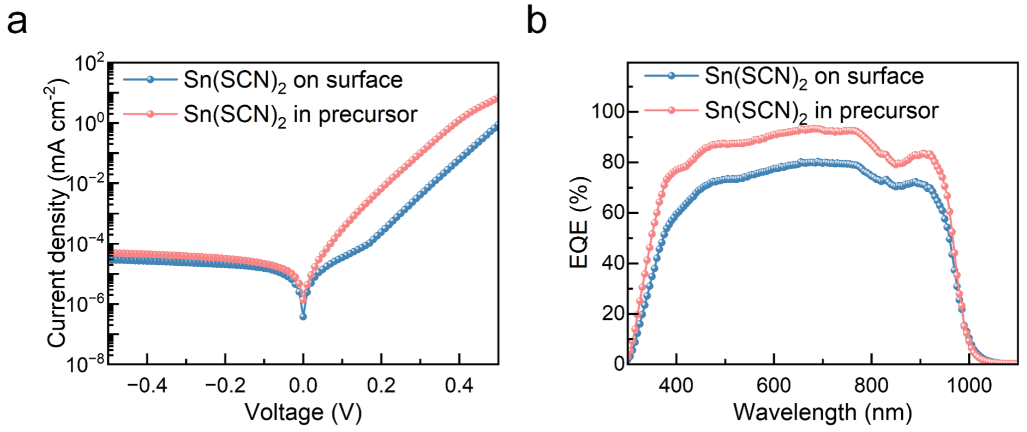

Fig. S9 | a Dark current density and **b** EQE spectra of Sn-Pb perovskite photodetectors incorporating $Sn(SCN)_2$ in precursor or on surface.

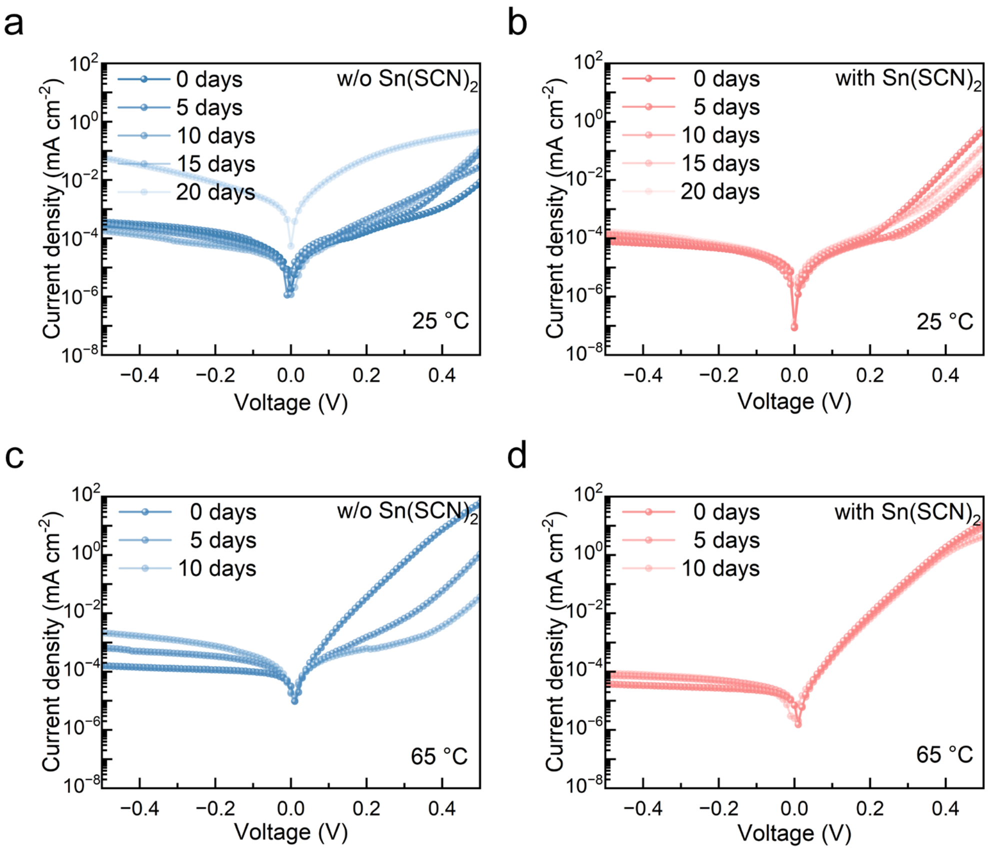

Fig. S10 | The stability of unencapsulated devices **a** without and **b** with $Sn(SCN)_2$ stored in a nitrogen glove box at 25 °C. The stability of unencapsulated devices **d** without and **d** with $Sn(SCN)_2$ stored in a nitrogen glove box at 65 °C.

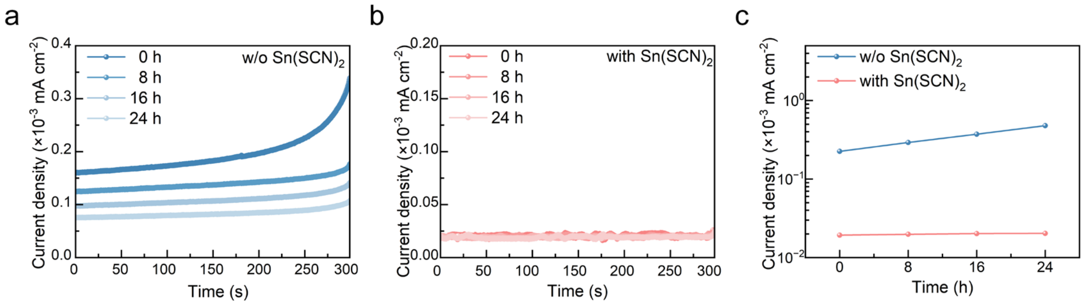

Fig. S11 | The stability of unencapsulated devices **a** without and **b** with $Sn(SCN)_2$ under continuous working at a bias of −0.1 V in a nitrogen glove box. **c** Comparison of dark current density for devices with and without $Sn(SCN)_2$ continuous working at a bias of −0.1 V in a nitrogen glove box.

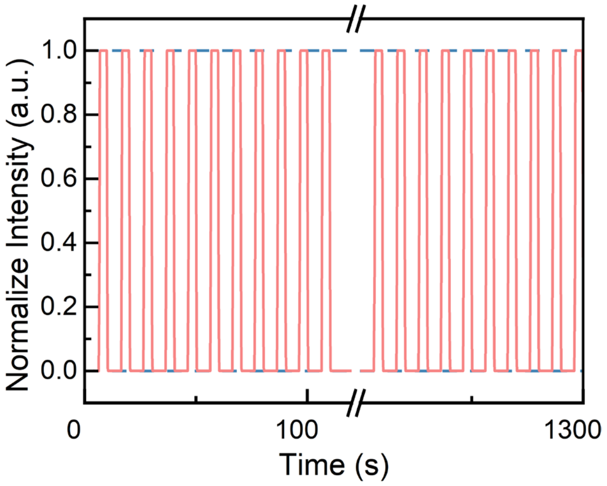

Fig. S12 | Continuous track of the photo and dark current of the Sn-Pb perovskite photodetector with $Sn(SCN)_2$ during a period of 1300 s.

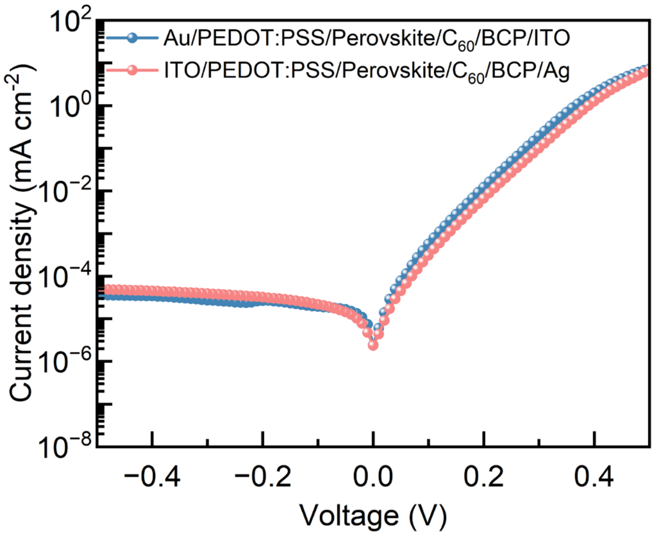

Fig. S13 | Dark current density versus voltage curves of Sn-Pb perovskite photodetectors with different top electrodes.

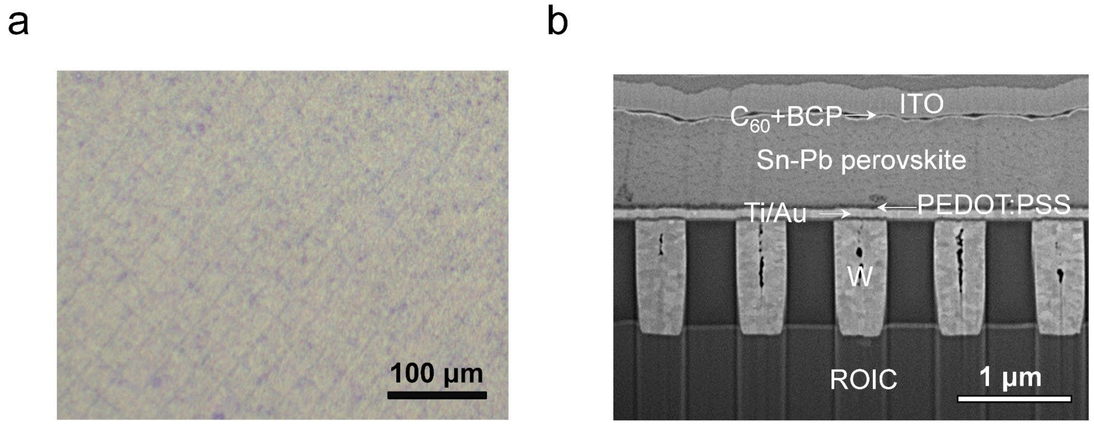

Fig. S14 | a Microscopic image of Sn-Pb perovskite photodiodes covering on the CMOS ROIC chip. **b** Cross-section SEM image of the Sn-Pb perovskite imager.

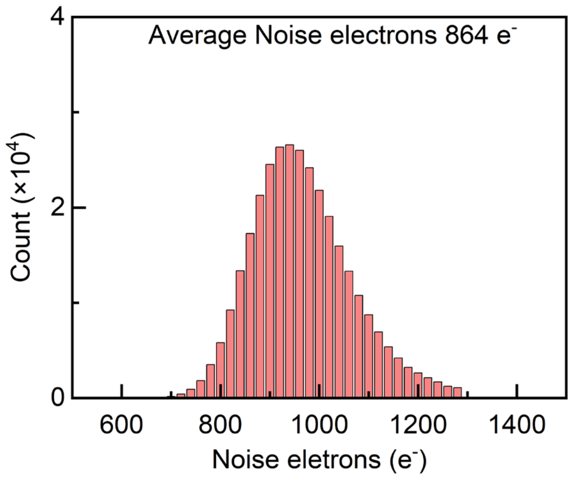

Fig. S15 | The statistical graph of the number of the noise electrons in the focal plane.

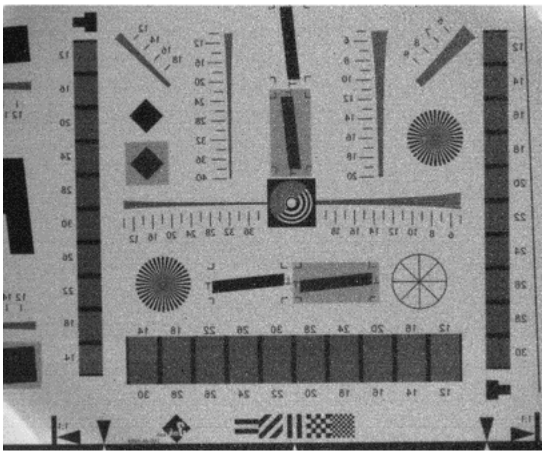

Fig. S16 | ISO-12233 test chart captured by the Sn-Pb perovskite imager without $Sn(SCN)_2$ under 940 nm light.

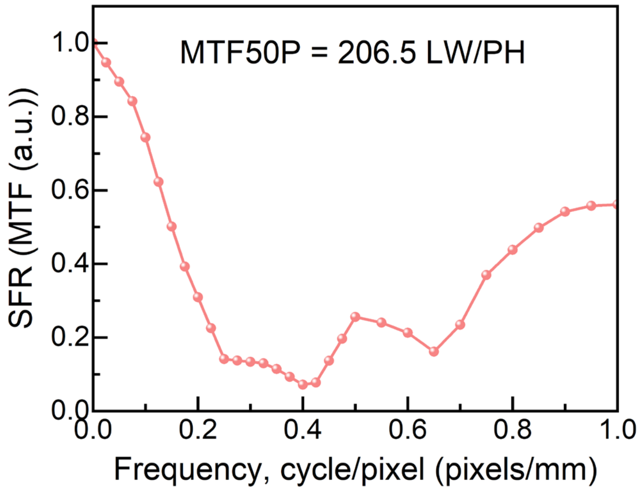

Fig. S17 | MTF of the Sn-Pb perovskite imager.

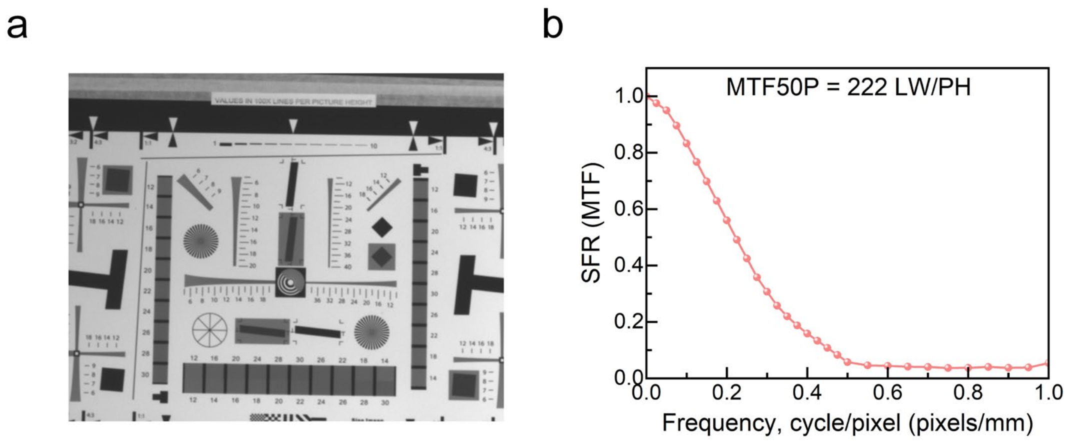

Fig. S18 | a ISO-12233 test chart imaged by the InGaAs imager (IMX991-AABA-C, 640 × 512 pixels). **b** The MTF of the InGaAs imager (IMX991-AABA-C, 640 × 512 pixels).

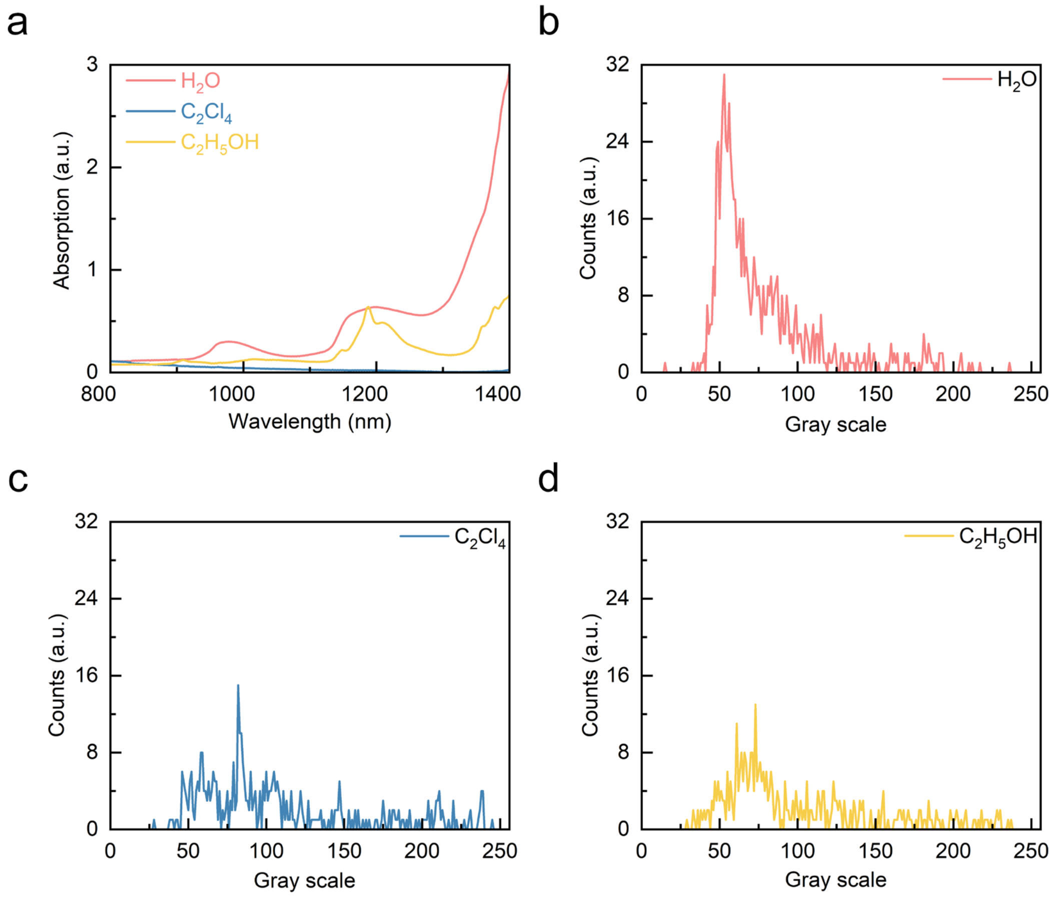

Fig. S19 | a Absorption spectra of H_2O, C_2Cl_4 and C_2H_5OH. The gray scale distribution of **b** H_2O, **c** C_2Cl_4 and **d** C_2H_5OH in Fig. 4h.

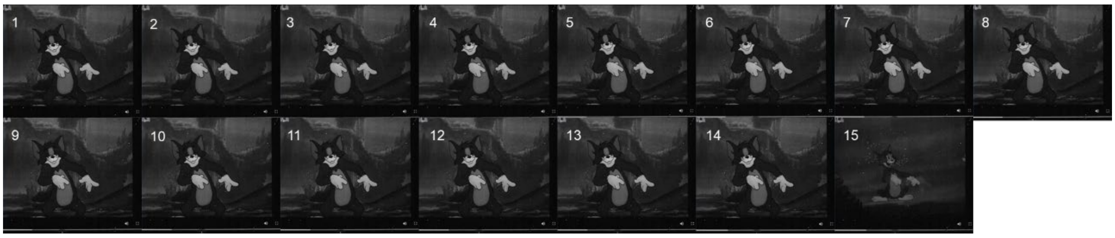

Fig. S20 | 15 frames in 7 s-7.5 s in the video in Supplementary Video 1.

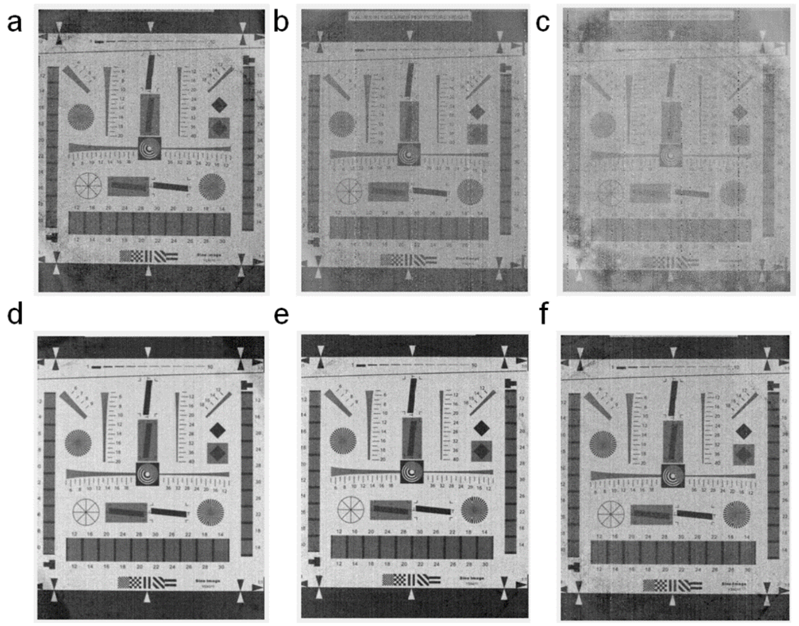

Fig. S21 | ISO-12233 test chart captured by the Sn-Pb perovskite imager without $Sn(SCN)_2$ under 940 nm light after stored in N_2 for **a** 0 day, **b** 7 days and **c** 14 days. ISO-12233 test chart captured by the Sn-Pb perovskite imager with $Sn(SCN)_2$ under 940 nm light after stored in N_2 for **d** 0 day, **e** 7 days and **f** 14 days.

Table S1 | Specific detectivity over 900 nm and dark current density at −0.1 V of Sn-Pb perovskite detectors reported in the literatures.

Year	Specific detectivity (Jones)	Dark current density ($mA\ cm^{-2}$)	Array size	References
2020	~2×10^{11}	1.25×10^{-3}	Single-pixel	5
2021	~2.5×10^{12}	~1.5×10^{-7}	Single-pixel	6
2022	~6.57×10^{11}	~6×10^{-5}	Single-pixel	7
2022	~1.5×10^{12}	~1.0×10^{-5}	Single-pixel	8
2023	~2.5×10^{12}	~9×10^{-4}	Single-pixel	9
2023	~1.56×10^{11}	~1×10^{-3}	Single-pixel	10
2023	~1×10^{12}	~1×10^{-3}	Single-pixel	11
2023	~8.48×10^{12}	~1.5×10^{-5}	Single-pixel	12
2023	~3×10^{12}	1.2×10^{-5}	5 × 5	13
2024	~3.4×10^{12}	-	2 × 2	14
2024	~8×10^{11}	6.27×10^{-5}	Single-pixel	15
2025	~1.6×10^{13}	1.0×10^{-5}	640 × 512	This work

References

1. Liu, J. et al. A near-infrared colloidal quantum dot imager with monolithically integrated readout circuitry. *Nat. Electron.* **5**, 443–451 (2022).

2. Rogalski, A. Optical detectors for focal plane arrays. *Opto-Electron.* **12**, 221-245 (2004).

3. Shekhar, H. et al. Hybrid image sensor of small molecule organic photodiode on CMOS – Integration and characterization. *Sci. Rep.* **10**, 7594 (2020).

4. Chen, L. et al. Complementary passivation of bidentate aromatic ligands enables high-temperature stable PbS colloidal quantum dots image sensor. *Adv. Funct. Mater.* 2501770 (2025) doi:10.1002/adfm.202501770.

5. Zhao, Y., Li, C., Jiang, J., Wang, B. & Shen, L. Sensitive and stable tin–lead hybrid perovskite photodetectors enabled by double-sided surface passivation for infrared upconversion detection. *Small* **16**, 2001534 (2020).

6. Ollearo, R. *et al.* Ultralow dark current in near-infrared perovskite photodiodes by reducing charge injection and interfacial charge generation. *Nat. Commun.* **12**, 7277 (2021).

7. Wang, Y. *et al.* Dual organic spacer cation quasi-2D Sn–Pb perovskite for solar cells and near-infrared photodetectors application. *Adv. Photonics Res.* **3**, 2200079 (2022).

8. Morteza Najarian, A. *et al.* Sub-millimetre light detection and ranging using perovskites. *Nat. Electron.* **5**, 511–518 (2022).

9. Liu, F. *et al.* Highly efficient and stable self-powered mixed tin-lead perovskite photodetector used in remote wearable health monitoring technology. *Adv. Sci.* **10**, 2205879 (2023).

10. Lv, Y., Cen, G., Li, W., Zhao, C. & Mai, W. Highly sensitive fast-response near-infrared photodetectors based on triple cation Sn-Pb perovskite for pulse oximetry system. *Sci. China Mater.* **66**, 4704–4710 (2023).

11. Zhao, R. *et al.* Highly efficient and stable near-infrared photodetectors enabled from passivated tin–lead hybrid perovskites. *Nanotechnology* **34**, 215702 (2023).

12. Liu, H. *et al.* Realizing high-detectivity near-infrared photodetectors in tin–lead perovskites by double-sided surface-preferred distribution of multifunctional tin thiocyanate additive. *ACS Energy Lett.* **8**, 577–589 (2023).

13. He, L. *et al.* Highly sensitive tin-lead perovskite photodetectors with over 450 days stability enabled by synergistic engineering for pulse oximetry system. *Adv. Mater.* **35**, 2210016 (2023).

14. Li, W. *et al.* The UV–vis-NIR broadband ultrafast flexible Sn-Pb perovskite photodetector for multispectral imaging to distinguish substance and foreign-body in biological tissues. *Adv. Opt. Mater.* **12**, 2301373 (2024).

15. Chen, H. *et al.* Stable and sensitive tin-lead perovskite photodetector for pulse oximetry sensing. *IEEE Electron Device Lett.* **45**, 1570–1573 (2024).
